# A case report of a sub-clinical necrotising lower limb infection secondary to pelvic anastomotic leak and chronic corticosteroid use

**DOI:** 10.1016/j.jpra.2019.03.004

**Published:** 2019-04-17

**Authors:** Jake Nowicki, Mariana Rego, Nicola R. Dean

**Affiliations:** Department of Plastic and Reconstructive Surgery, Flinders Medical Centre, Bedford Park 5041, Adelaide, South Australia, Australia

**Keywords:** Corticosteroids, Bowel perforation, Sciatic notch, Diverticulitis, Leg abscess

## Abstract

Anabolic steroids have attracted attention from the media with regard to misuse, but overuse of medically prescribed steroids also has a propensity to cause harm. Gluteal necrotising soft tissue infections are an uncommon presentation for plastic surgeons, and therefore, abdominal sources may be overlooked. We report a rare case of a 57-year-old male who presented with a necrotising lower limb infection on a background of long-term corticosteroid overuse and recurrent pelvic anastomotic leaks.

## Case report

A 57-year-old male presented to hospital with right hip pain and an inability to mobilise, which were attributed to a mechanical fall four days earlier. The patient's medical history included a Hartmann's procedure for a colovesical fistula and perforated diverticular disease, with a subsequent failed reversal secondary to colonic ischaemia and anastomotic leak at the rectosigmoid anastomosis. An ileorectal anastomosis was created at the second Hartmann's reversal. This was further complicated by an enterocutaneous fistula from the small bowel to the umbilicus. Nine months prior to admission, he had been prescribed daily prednisolone (10 mg) by his GP to assist with poor appetite. For the preceding three months, he had increased his daily dose to 20 mg with unclear indication and not under supervision of a doctor.

**Figure 1:**
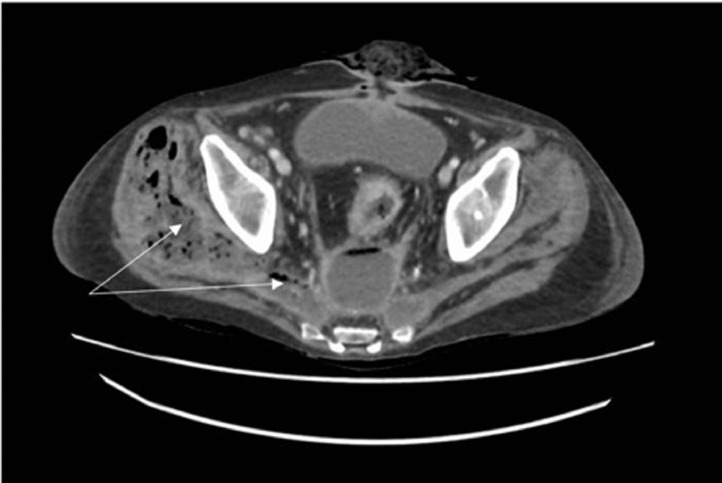
Computed tomography of the patient at presentation to hospital. Arrows indicate to gas observed in the gluteal soft tissues, which tracks into the pelvis. Communicating gluteal collection and sacral collection are also observed and highlighted.

On presentation, the patient was afebrile but hypotensive at 92/60 mmHg. The area over the right greater trochanter was tender to deep palpation, oedematous and discharging, with flexion and extension limited by pain. His abdomen was non-tender but demonstrated a mid-line point of discharge consistent with an enterocutaneous fistula. Blood tests revealed an elevated C-reactive protein level of 140 mg/mL and a white cell count of 11.0 × 10^9^/L. A plain radiograph of the hip showed lateral subcutaneous emphysema. Computed tomography (CT) scan of the abdomen and pelvis showed communicating pre-sacral and gluteal fluid collections, with gas in the soft tissues of the gluteal region and posterior thigh (Figure 1). The pre-sacral collection had persistently been observed on CT because of his ileorectal anastomosis; however, the gluteal collection was new.

**Figure 2:**
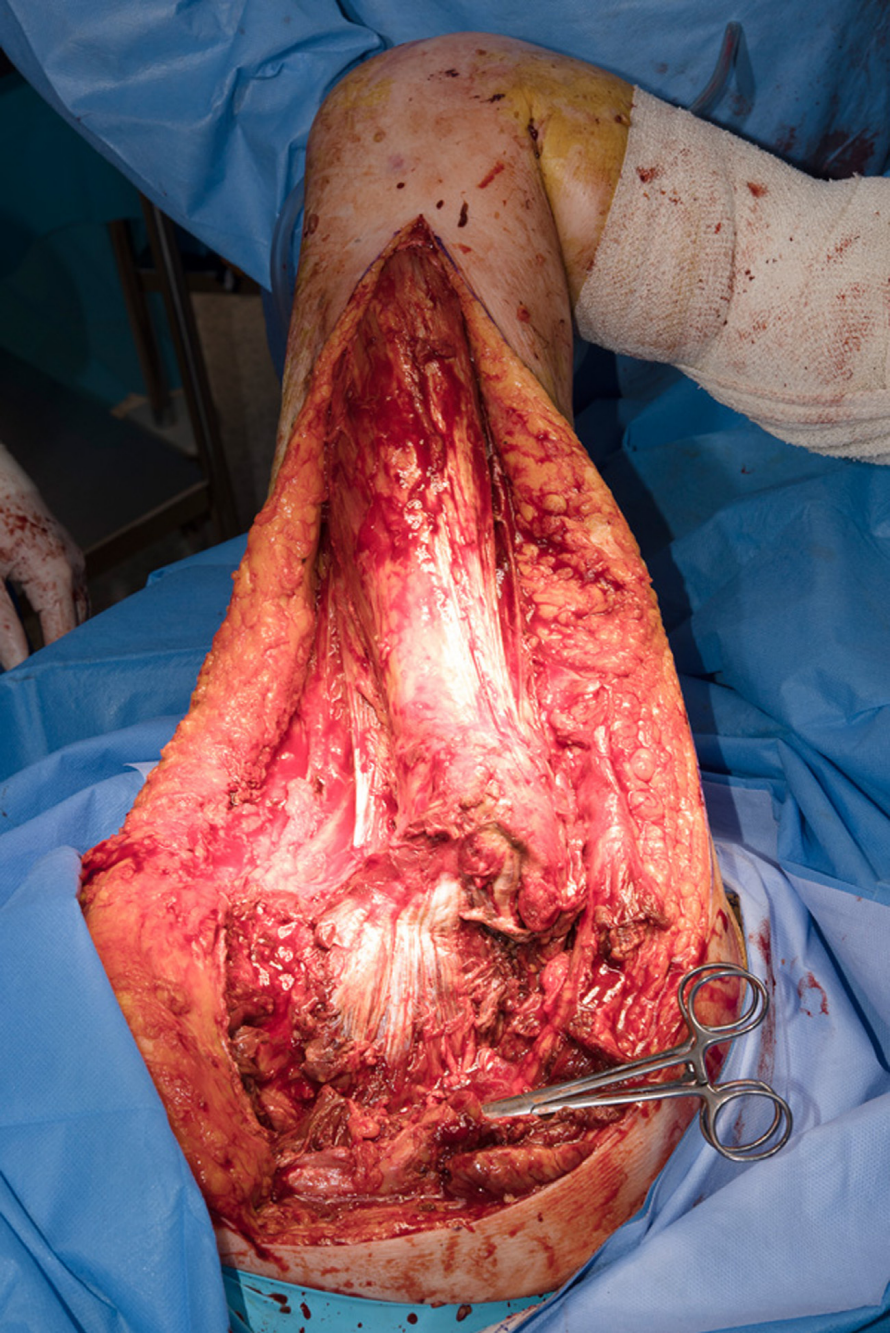
Intraoperative photo during first procedure in the patient. Gluteus maximus and medius and tensor fascia lata have been debrided, with gluteus minimus visible in the photo. The thigh musculature is intact and did not require debridement.

The patient was reviewed by the Department of Plastic Surgery and General Surgery and taken to the operating theatre within one hour of referral. Pus was found in the gluteal region with a communicating tract into the sacral notch. Gluteus maximus and medius and tensor fascia lata were necrotic and debrided (Figure 2). Urgent microscopy demonstrated gram-positive cocci and bacilli as well as gram-negative bacilli. Microbiological cultures later yielded extended spectrum beta-lactamase *Escherichia coli* and *Clostridium sporogenes.* The anterior and posterior compartments of the thigh were assessed and had healthy viable tissue with no pus. A laparotomy was performed during the same procedure by the colorectal surgeons. The pre-existing enterocutaneous fistula was found to be communicating with an entero-enteric fistula involving three loops of small bowel communicating with each other in the right lower quadrant. The distal end of the entero-enteric fistula tracked to the ileo-rectal anastomosis. Fistulotomies were completed, and the involved small bowel loops were resected. An end-ileostomy was created, and the rectal stump was oversewn.

**Figure 3:**
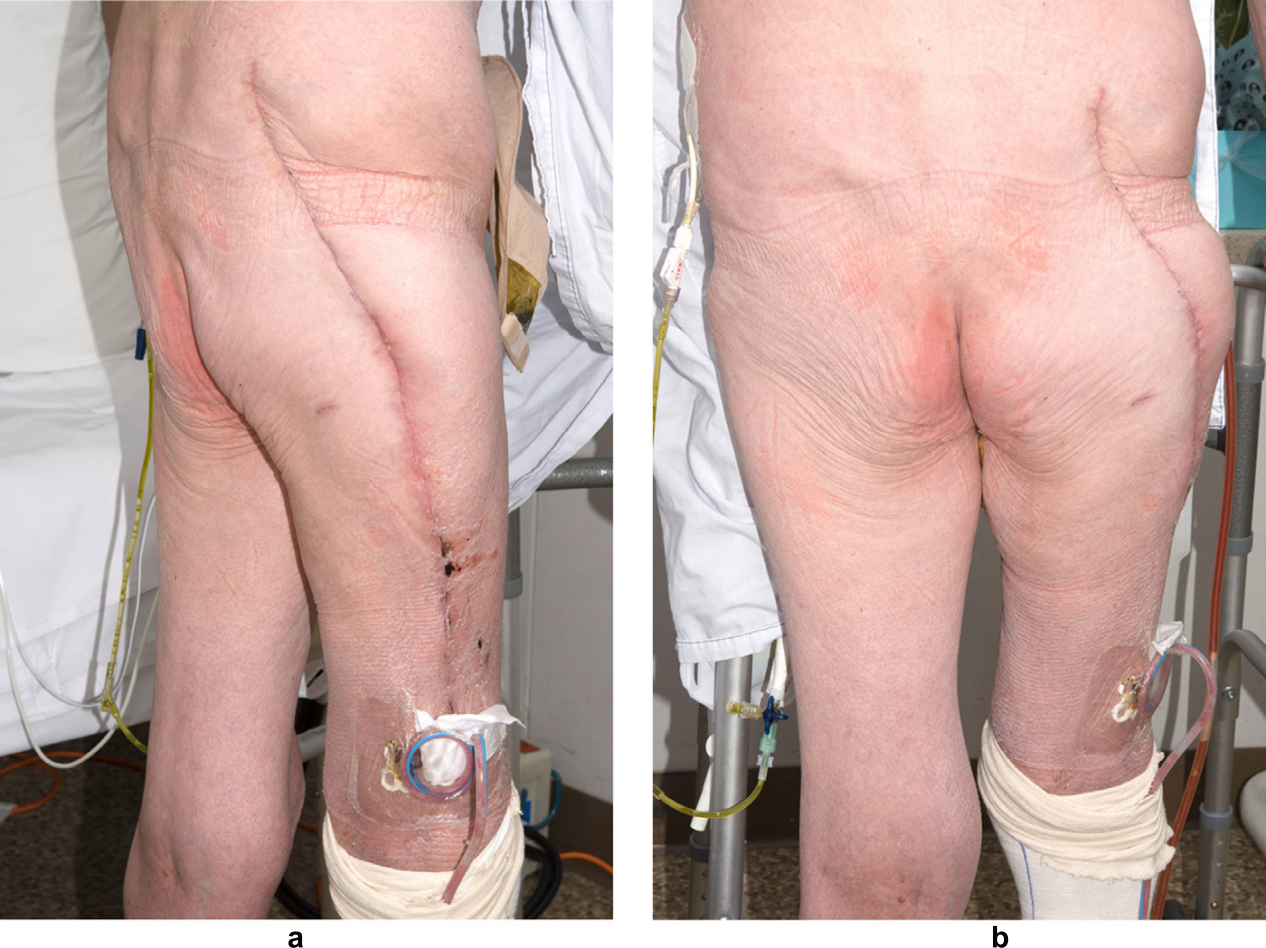
(a) (left) and (b) (right). Patient's wounds after one month of admission. Note the patient had protracted wound healing likely secondary to malnutrition and corticosteroid-induced immunosuppresion.

The patient returned to theatre the following day, and necrotic piriformis was debrided. Nine days later, he exhibited increased serum inflammatory markers and had brown discharge from his laparotomy wound. CT demonstrated multiple new abdominal collections in the lumbar, paraumbilical, and iliac regions, which underwent percutaneous CT-guided drainage. His wounds healed slowly, likely secondary to chronic corticosteroid induced immunosuppression and malnutrition (Figure 3). An adenocorticotrophic hormone stimulation test demonstrated adrenal suppression, which required slow weaning of his steroids. The patient was discharged to a rehabilitation centre after two months in hospital.

One week post-discharge, he was referred back to hospital by his community wound nurses because of increasing erythema and swelling of his right thigh wound. He was taken to theatre where an extensive subcutaneous abscess was drained from his gluteal and posterior thigh regions. Eighteen months later, the patient was re-admitted with sepsis and found to have a loculated pre-sacral abscess communicating with the right thigh collection, requiring further surgical washout. He has been subsequently undergoing follow-up in colorectal and plastic surgery outpatient clinics.

## Discussion

Anabolic steroids have attracted much attention from the media with regard to misuse,^1^ but overuse of any type of steroids has a propensity to cause harm. Corticosteroids are an invaluable class of medication used for treatment of a range of medical conditions; however, it is well documented that its long-term use is associated with significant side effects, including osteoporosis, metabolic disease, and cardiovascular disease.^2^, ^3^ Corticosteroids are also immunosuppressive and predispose patients to potentially devastating infections.^4^ The prevalence of abuse of prescribed corticosteroids is not well documented in the literature. Motivation for overuse may be the euphoric effects of corticosteroids along with a lack of understanding of their health risk.

Corticosteroid misuse was the likely reason that this patient presented at such a late stage exhibited a lack of clinical signs such as pyrexia and wound erythema. Similar case reports have described patients on long-term corticosteroid therapy suffering infections in the lower limb tracking from the abdomen,^5^, ^6^ but there has been limited description of their surgical management and outcomes. In our case, the patient avoided disarticulation at the hip but required debridement of several necrotic hip muscles.

Failure of colorectal anastomosis can have dire consequences, both acute and long term, and is associated with increased morbidity and mortality.^7^ Leak rates vary from 6% to 30% depending on risk factors and varying definitions of anastomotic leak.^7^ The proportion of pelvic leaks that track into the buttock is not documented in the literature. Previous reports have documented necrotising fasciitis of the thigh secondary to colonic perforation,^5^, ^8^, ^9^ but all reported patients presented with coexisting abdominal symptoms. Only one report described tracking of the infection through the sciatic notch, with the others reported spread through the femoral canal.^9^ Diverticulitis has been reported as the cause of colonic perforation, leading to infection of the lower limb in two cases.^5^, ^10^

Several points are highlighted by this rare case. Plastic surgeons when seeing patients with necrotising infections of the gluteal and thigh regions should be alert to the possibility of an intra-abdominal source, especially in those whose immune response may be blunted by corticosteroid use. Patients on long-term corticosteroids should be counselled on their significant adverse effects.
